# Optimized Expression and Purification for High-Activity Preparations of Algal [FeFe]-Hydrogenase

**DOI:** 10.1371/journal.pone.0035886

**Published:** 2012-04-26

**Authors:** Iftach Yacoby, Lotta Tollstoy Tegler, Sergii Pochekailov, Shuguang Zhang, Paul W. King

**Affiliations:** 1 Center for Biomedical Engineering, Massachusetts Institute of Technology, Cambridge, Massachusetts, United States of America; 2 Biosciences Center, National Renewable Energy Laboratory, Golden, Colorado, United States of America; New England BioLabs, United States of America

## Abstract

**Background:**

Recombinant expression and purification of metallo-enzymes, including hydrogenases, at high-yields is challenging due to complex, and enzyme specific, post-translational maturation processes. Low fidelities of maturation result in preparations containing a significant fraction of inactive, apo-protein that are not suitable for biophysical or crystallographic studies.

**Principal Findings:**

We describe the construction, overexpression and high-yield purification of a fusion protein consisting of the algal [2Fe2S]-ferredoxin PetF (Fd) and [FeFe]-hydrogenase HydA1. The maturation of Fd-HydA1 was optimized through improvements in culture conditions and media components used for expression. We also demonstrated that fusion of Fd to the N-terminus of HydA1, in comparison to the C-terminus, led to increased expression levels that were 4-fold higher. Together, these improvements led to enhanced HydA1 activity and improved yield after purification. The strong binding-affinity of Fd for DEAE allowed for two-step purification by ion exchange and StrepTactin affinity chromatography. In addition, the incorporation of a TEV protease site in the Fd-HydA1 linker allowed for the proteolytic removal of Fd after DEAE step, and purification of HydA1 alone by StrepTactin. In combination, this process resulted in HydA1 purification yields of 5 mg L^−1^ of culture from *E. coli* with specific activities of 1000 U (U = 1 µmol hydrogen evolved mg^−1^ min^−1^).

**Significance:**

The [FeFe]-hydrogenases are highly efficient enzymes and their catalytic sites provide model structures for synthetic efforts to develop robust hydrogen activation catalysts. In order to characterize their structure-function properties in greater detail, and to use hydrogenases for biotechnological applications, reliable methods for rapid, high-yield expression and purification are required.

## Introduction

The direct conversion of low potential electrons into hydrogen by microbial organisms is a prospective biotechnological process for the renewable hydrogen production as an energy carrier [1], [2], [3]. Photosynthetic microbes, for example green algae [4], [5], [6] and cyanobacteria [7], can couple light-driven water oxidation to generate hydrogen, whereas anaerobes produce hydrogen utilizing the reduced electron carriers generated by fermentation [8], [9]. In both examples, the hydrogen producing reactions are catalyzed by hydrogenases. Hydrogenases catalyze hydrogen activation by virtue of unique organometallic clusters that compose the catalytic sites [10], [11]. Based on the metal compositions of the catalytic sites, the hydrogenases organize into phylogenetic groups representing three enzyme subclasses [11], [12]. These are; (i) [NiFe]-hydrogenases found in cyanobacteria, archaea and eubacteria; (ii) iron-sulfur-cluster-free hydrogenases, for example HMD hydrogenase from the methanogen *Methanothermobacter marburgensis* [13], [14]; and (iii) [FeFe]-hydrogenases found in anaerobic eubacteria and protozoans, and green algae. The [FeFe]-hydrogenases, which are the focus of this investigation, generally exhibit a catalytic bias towards hydrogen production, possess high *k*_cat_ values, monomeric compositions and show high sensitivity to oxygen [3]. Because of these properties, [FeFe]-hydrogenases, as well as [NiFe]-hydrogenases, are being investigated to understand the structural basis for both fast hydrogen activation rates [15] and for oxygen tolerance [14], [16] towards achieving efficient natural and artificial solar hydrogen production systems [17], [18], [19].

In recent years, several groups have developed different strategies for the recombinant expression of [FeFe]-hydrogenases [20] (see Table 1). These include using either native [FeFe]-hydrogenase expressing organisms as hosts [21], [22], or *Escherichia coli* for heterologous expression. Initial expression efforts in *E. coli* relied on co-expression of the required maturation genes HydE and HydFG from *Chlamydomonas reinhardtii* [23]. Due to the instability of the *C. reinhardtii* genes in *E. coli*, an expression system was developed utilizing the HydE, HydF and HydG maturation genes along with the HydA structural gene from *Clostridium acetobutylicum* [24]. A similar approach was also used for co-expression of the *Shewanella oneidensis* maturation genes in *E. coli* where improvements in growth conditions resulted in higher expression levels and recovery yields of 30 mg L^−1^ of culture [25]. However, the maturation efficiencies based on the Fe content and specific activities indicated H-cluster incorporation was not complete. In addition, the purification of recombinant hydrogenases is most often performed by use of affinity tags, for example His or StrepII-tag, at the N- or C-terminus [21], [22], [24], [25], [26]. In most cases affinity purification is performed as a single-step process that typically results in rather low yields, and purities at or greater than 80%. Although suitable for biochemical characterization, both crystallization and biophysical studies require production of large quantities of samples that are also high in purity and quality.

**Table 1:** HydA1 purification yields and specific activities from various expression hosts.

Host	Specific activity^a^	Total yield^b^	Iron content^c^	Ref
*C. reinhardtii*	730	0.04	NR	[33]
*C. reinhardtii*	935	0.001	3.9±0.3	[34]
*S. oneidensis*	740	0.5	6.1±0.1	[22]
*C. acetobutylicum*	625	1	NR	[30]
*C. acetobutylicum*	760	0.1	NR	[21]
*E. coli*	150	1	NR	[24]
*E. coli*	641	30	4.5±0.2	[25]
*E. coli*	400	1.5	NR	[26]
*E. coli*	1000	5	5.64±0.28	Current work

a HydA1 specific activities for hydrogen evolution by MV assay; 1 U = µmol hydrogen min^−1^ mg^−1^.

b HydA1 mg L^−1^ of cell culture.

c Iron content as mol iron (mol HydA1)^−1^; NR, not reported.

Recently, we have reported on the design and expression of a fusion protein composed of the [2Fe2S] Fd PetF and the [FeFe]-hydrogenase HydA1 from *C. reinhardtii* [26]. Here, we show that the addition of the Fd as a fusion partner to HydA1 facilitated higher expression and purification yields of HydA1 [FeFe]-hydrogenase. These effects were observed when Fd was fused to the N-terminus rather than the C-terminus of HydA1. Moreover, by incorporating a TEV protease site in the fusion linker it was possible to remove the Fd prior to the final affinity step, resulting in pure HydA1. After this final step, HydA1 preparations possessed >95% Fe incorporation and specific activities of 1000 U (U = 1 µmol hydrogen mg^−1^ min^−1^) for hydrogen evolution from reduced methyl viologen (MV). In summary, the Fd fusion based expression process improved both the yields and maturation fidelities of recombinant [FeFe]-hydrogenase HydA1 production in *E. coli*.

## Results

### Construction of Fd-HydA1 fusion protein with a cleavable TEV linker

**Figure 1:**
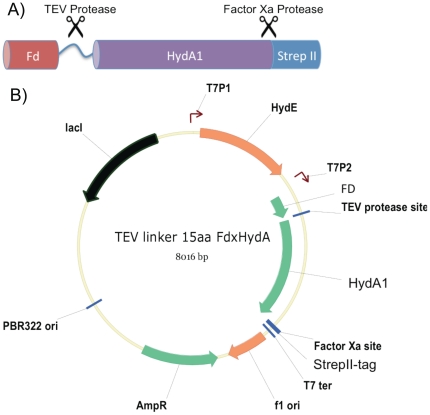
Diagrams of the Fd-HydA1 fusion protein and the pHydEFd-HydA1 expression plasmid developed for the study. (A). A schematic of the N-terminal Ferredoxin-HydA1 fusion protein showing the relative positions of Ferredoxin, (Fd) peptide linker with TEV protease site (TEV protease), HydA1 and the Factor XA Protease and StrepII-tag (Strep II). HydA1 (*C. reinhardtii HYDA1*) is the mature protein sequence (without the chloroplast transit peptide). (B). The pHydEFd-HydA1 plasmid is a derivative of pETDuet (Invitrogen, USA) that allows for IPTG-inducible co-expression of the HydE and Fd-HydA1 proteins [11]. The relative locations of HydE, Fd, TEV protease site, HydA1, Factor Xa protease site and the StrepII tag are shown. T7P1, T7 polymerase promoter 1; T7P2, T7 polymerase promoter 2; T7ter, T7 transcriptional terminator; AmpR, ampicillin resistance marker; pBR322 ori, origin of replication; and LacI, lactose repressor gene.

For this work, an Fd-HydA1 fusion was created that incorporated two unique protease sites, one for proteolytic cleavage of Fd from HydA1 and one for removal of the HydA1 C-terminal StrepII-tag. The peptide linker that connects Fd to HydA1 incorporated the TEV protease site ENLYFQ(G/S) [27], [28], and the HydA1 C-terminus includes a Factor Xa cleavage followed by the StrepII-tag (Figure 1A). This construct was sub-cloned into the T7 expression plasmid pHydE [24], which harbors the maturation protein *C. acetobutylicum* HydE gene, to create pHydEFd-HydA1 (Figure 1B). Both pHydEFd-HydA1 and plasmid pHydFHydG [11] were co-transformed into *E. coli* BL21 (DE3) Rosetta-2, which in the presence of IPTG co-expresses the three maturation proteins, HydE, HydF and HydG, together with Fd-HydA1.

### Optimization of culture conditions for recombinant [FeFe]-hydrogenase expression

This recombinant expression of [FeFe]-hydrogenases in *E. coli* is a multi-step process involving use of temperature shifts, differential gas-phase conditions, and culture vessels [11]. An extensive screen of each of these parameters, together with media and antibiotic formulations was performed to improve [FeFe]-hydrogenase expression yields and activities using Fd-HydA1 for testing. Terrific broth (TB) was used as the base media formulation due its use for high-level expression of Fd [29]. This was confirmed by comparing HydA1 expression yields in TB to those from cells grown in LB or minimal media. Cultures grown in TB consistently produced significantly higher HydA1 yields, thus TB was selected for optimization of additional growth factors and conditions. For HydA1 and Fd-HydA1 expression, cultures are grown in four steps; the first three were performed under aerobic conditions and the last under anaerobic conditions, described as follows:

- Day 1. An overnight 10 ml aerobic starter culture (ON).
- Day 2. Small aerobic starter culture. A 1∶100 dilution of the ON into 10 ml of fresh media (SD1).
- Day 2. Large aerobic large culture. A 1∶100 dilution of SD1 into 1 L of fresh media (SD2).
- Days 2 and 3. Anaerobic expression culture. The SD2 is transferred to a gas-tight, anaerobic bottle, equilibrated under argon for 1 h and incubated overnight under an H_2_/N_2_ atmosphere. (AC)

**Figure 2:**
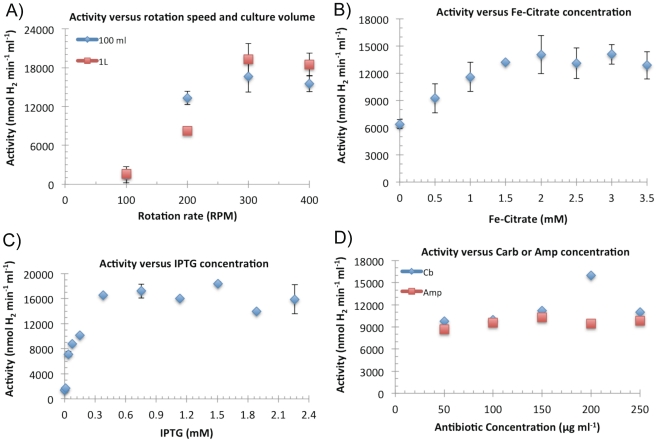
Influence of growth factors on the *in vitro* hydrogen evolution activities in cells harboring pHydEFd-HydA1 and pHydFHydG. Hydrogenase activities (reported in units of nmol hydrogen evolved, ml^−1^ of culture, min^−1^) in solubilized whole-cells were measured as described in materials and methods. (A). The effect of the shaker rotation speed (RPM) during the aerobic growth phase was tested for 100 ml or 1 L culture volumes grown in. 250 ml or 2 L baffled flasks, respectively. The optimal rate was for both culture volumes was ∼300–350 RPM. (B). The effect of ferric ammonium citrate (FEC) concentration. Optimal levels were 2–2.5 mM. (C). Effect of IPTG concentration. Optimal levels of IPTG were 0.4 mM, lower than the 1.5 mM used previously [13]. (D). Effect of substituting Ampicillin with Carbenicillin during all stages of growth. Addition of Carbenicillin at 200 µg ml^−1^ was required for peak hydrogenase activity levels.

The first culture parameter that was tested for optimization was the aeration rate during growth of SD2. While common expression protocols suggest 200–250 rpm shaking, we found that a more rapid rotation rate of 300–350 rpm resulted in 2-fold higher Fd-HydA1 expression levels, based on whole-cell activity assays (Figure 2A). Since HydA1 and Fd are both iron-sulfur proteins, we also tested the effect of different iron supplementation levels used during AC on whole-cell activities. A concentration range of ferric ammonium citrate (FEC) was added at the start of the AC phase at the same time as the addition of IPTG. Based on the activity levels, the optimal value of FEC was 2–3 mM final (Figure 2B). The effect of IPTG concentration was also tested and found to be optimal at levels of only 0.4 mM (Figure 2C). Two types of antibiotics are required to stably maintain the expression plasmids; Streptomycin (Str) (pHydFHydG) and ampicillin (Amp) for the pHydEFd-HydA1 (Figure 1B). While Str is stable under culture conditions, Amp is gradually degraded by accumulation of extracellular β-lactamases during growth. When Amp was replaced with Carbenicillin (Carb), which is less easily degraded by β-lactamases, expression levels increased up to 2-fold at 200 µg ml^−1^ (Figure 2D). Interestingly, we found that the presence of antibiotic is crucial only for the ON and SD1 steps, whereas if the SD2 and AC steps could be performed in the absence of antibiotics, with no loss in Fd-HydA1 yields (data not shown). When the culture modifications were combined into a single process, the hydrogenase yields and whole-cell activity levels increased ∼10-fold, from 0.5 mg L^−1^ of culture at 2 µmol hydrogen ml^−1^ min^−1^ to 5 mg L^−1^ of culture at 20 µmol hydrogen ml^−1^ min^−1^. In comparison to our previous studies [13], the improvement led to 3-fold increase in purification yield from 1.5 to 5 mg L^−1^ of culture, and a 6-fold increase in whole-cell activities from 3.5-to 22 µmol hydrogen ml^−1^ min^−1^.

### Two-step purification of Fd-HydA1

**Figure 3:**
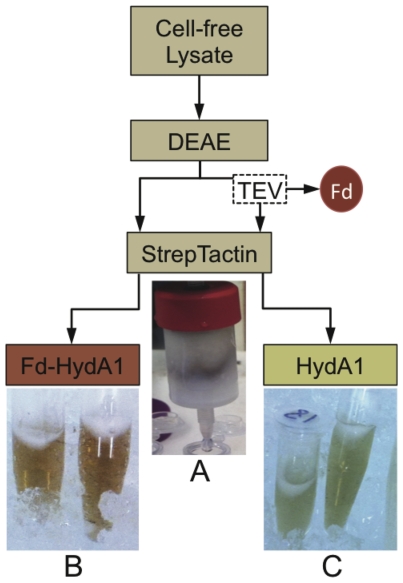
Illustrated purification steps. As shown in the schematic, clarified, cell-free lysates were separated on DEAE ion-exchange and the pooled hydrogenase active fractions were separated on StrepTactin columns. (A). During elution from StrepTactin, a distinct brown band was evident that contained the StrepII tagged HydA1. (B). Purified Fd-HydA1, showing the intense red-brown coloration due to the addition of Fd, after single-step elution from StrepTactin in buffer containing 12.5 mM desthiobiotin and 500 mM NaCl. (C). TEV proteolytic removal of Fd after the DEAE step resulted in a color change from red-brown to green-brown of the purified HydA1.

Once expression and maturation were optimized, a new purification protocol was developed to optimize both yields and activities of Fd-HydA1. This involved a two-step process of ion exchange chromatography by DEAE followed by affinity chromatography with StrepTactin. After cell disruption and clarification, the soluble fraction was loaded directly onto a series of four pre-packed 5 ml DEAE columns. The high, overall negative charge of Fd enabled the tight binding of Fd-HydA1 to DEAE, and the Fd-HydA1 enriched fraction was eluted in a single step with 500 mM NaCl. Due to the additional presence of Fd, the sodium dithionite (NaDT)-reduced Fd-HydA1 fusion was a clear brown color, which enabled monitoring of protein elution visually without the need for a UV-Vis spectrometer (Figure 3A). The presence of Fd-HydA1 in each step of purification, from lysate preparation to column loading and elution, is evident by the dark brown color (see Figure 3A). Once Fd-HydA1 was purified (Figure 3B), the Fd moiety was removed by proteolytic cleavage using MBP-TEV as described in the Methods Section. Separation of HydA1 from the TEV digestion mixture was performed by StrepTactin affinity chromatography (Figure 3C). For this step it was critical to use high concentrations (1 M) of NaCl in the StrepTactin buffers, the use of lower NaCl concentrations resulted in the loss of HydA1 during loading. Even with the addition of 1 M NaCl, there was considerable migration of the bound HydA1 on the resin during the washing step, which resulted in a loss in the HydA1 yields. To overcome this, four 5 ml StrepTactin columns were combined in series to increase the bed volume. Elution of the pure HydA1 was performed using desthiobiotin and was evident as a green-brown enriched HydA1 fraction (Figure 3C).

### The N-terminal location of Fd enhances expression of the Fd-HydA1 fusion

**Figure 4:**
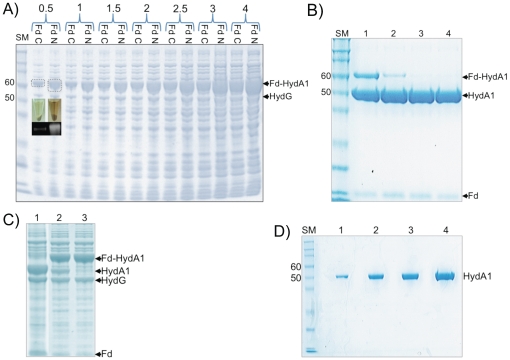
SDS-PAGE analysis of Fd-HydA1 expression, TEV digestion and HydA1 purification. (A). A comparison of the expression levels of N-terminal and C-terminal Fd-HydA1 fusions by separation on SDS-PAGE. Lanes show (left to right) protein size marker, SM, with sizes in kDa, and increasing amounts (0.5-to-4 µl) of total protein from cells expressing either the C-terminal (Fd C) or N-terminal (Fd N) Fd-HydA1 fusions. The location of ∼60 kDa Fd-HydA1 fusion, and 53 kDa HydG as an internal loading control are marked. The difference of Fd position on Fd-HydA1 expression levels was evident as a more intense brown color of the cell-lysates (inset image, top) harboring the N-terminal (right) versus C-terminal (left) Fd fusion. A Western Blot performed with antibodies to the StrepII-tag is shown (inset image, bottom) where the N-terminal fusion (right) exhibits a more intense band than the C-terminal fusion (left). (B). Analysis of TEV digestions of the N-terminal Fd-HydA1 fusion (30 µg) mixed with 1, 2, 4, or 8 µg of TEV (lanes 1–4, respectively). Lanes 2 and 3 show a partial digestion. The locations of Fd-HydA1 (60 kDa), HydA1 (50 kDa) and Fd (10 kDa) are indicated on the right. The optimal amount of TEV for complete digestion was 4 µg (shown in lane 3). Protein size-marker, SM, with sizes in kDa. (C). TEV digestions of DEAE pooled fractions containing Fd-HydA1 and separated on SDS-PAGE. The same w/w ratio of TEV to Fd-HydA1 was used as for purified Fd-HydA1 (4 µg TEV with 30 µg of DEAE pool). Lane 1, complete TEV digest; Lane 2, partial TEV digest; Lane 3, no TEV. Bands corresponding to Fd-HydA1 (60 kDa), HydA1 (50 kDa) and Fd (10 kDa) are identified on the right. HydG is identified as a loading control. (D). Analysis of HydA1 purity by SDS-PAGE. Protein size marker, SM, with sizes in kDa. Lanes 1–4 contain 1.4, 4, 6.8, and 12.3 µg, respectively, of purified HydA1 at >99% purity.

To determine how Fd contributed to the enhancement of hydrogenase activity levels in whole-cells expressing Fd-HydA1, we first compared the expression levels of N-terminal and C-terminal Fd fusions. As shown in Figure 4A, after equal loading of total cell protein, the N-terminal Fd fusion showed higher expression levels than those of the C-terminal Fd fusion. This was also evident from comparing clarified cell lysates containing N-terminal (Figure 4A, inset image right) or C-terminal (Figure 4A, inset image left) fusion. This difference was confirmed by separation of equivalent amounts of soluble fractions by SDS-PAGE and Western Blot with antibodies against the StrepII-tag (Figure 4A lower inset). The levels of the N-terminal fusion (right) are several times higher than the C-terminal fusion (left). Interestingly, the whole-cell MV activities are consistent with the expression results where the average cell-free extract activities for the N-terminal Fd fusion were 22 µmol hydrogen ml^−1^ min^−1^ compared to 7 µmol hydrogen ml^−1^ min^−1^ for C-terminal Fd-HydA1 at an equivalent OD_600_ value. In summary, the results indicate that the total HydA1 expression levels are elevated by 300–400% due to the presence of the N-terminal versus C-terminal location of Fd.

### Optimization of TEV digestion of Fd-HydA1

To determine the accessibility of the TEV recognition site in Fd-HydA1 for digestion, Fd-HydA1 was first purified by DEAE and StrepTactin chromatography. Different amounts of commercial TEV (TurboTEV, Eton Bioscience) were added to a fixed amount (30 µg) of Fd-HydA1 and the digestion was performed for 1 h at 37°C. We found that 4 µg of TEV or a w/w ratio of 1∶7.5 TEV∶Fd-HydA1 was sufficient for complete digestion (Figure 4B). The same TEV∶Fd-HydA1 ratio was also shown to result in complete digestion of fusion-enriched fractions from the DEAE purification step (Figure 4C). We repeated the digestion procedure using purified TEV-MBP and obtained similar results, thus TEV-MBP was used for the purification and analysis of HydA1 described below.

### Analysis of purified HydA1

We were able to obtain high purity HydA1 at concentrations of ∼2.5 mg ml^−1^ directly from StrepTactin columns by performing the TEV-MBP proteolytic digestion step after the DEAE chromatography. The purity was evaluated by SDS-PAGE and coomassie blue-staining (Figure 4D). The measured specific activity of the HydA1 in the MV activity assay was 1000 U, which compares well to previously published results [30]. It should be noted that after a freeze and thaw (e.g. when stored anaerobically at −80°C) the specific activity (MV) of pure HydA1 decreased from 1000 U to 300 U. Currently, we have no explanation for this observed loss of HydA1 MV activity following enzyme storage (see discussion below). However, this specific activity level of frozen stocks remained stable at 300–400 U over a period of several months at −80°C. The iron contents of purified HydA1 were determined as previously described [31], and found to be on average 5.64 (+/−0.28) mol iron (mol HydA1)^−1^, which indicates 94% incorporation based on 6 atoms of iron per HydA1. Overnight digestion of HydA1 with Factor Xa resulted in complete removal of the StrepII-tag.

## Discussion

The heterologous expression of metallo-enzymes, specifically hydrogenases, requires optimization of both protein expression and maturation processes, and each process can significantly affect the final production levels and enzyme qualities. In this study, we described an optimized protocol for recombinant production of [FeFe]-hydrogenase in *E. coli* developed from expression of an Fd-HydA1 fusion. We found that the major factors that contributed to increased expression levels were conditions that maintained more aerobic growth (i.e., baffled flasks and rotation rates >350 rpm), and the utilization of Carbenicillin in place of Ampicillin at the start of anaerobic expression phase. Together these two factors resulted in a 3-fold elevation in HydA1 yields compared to our previous report [13], or an increase from 1.5 to 5 mg L^−1^ of culture. It should be noted that it is possible that the expression protocol can be optimized even further from optimization of other growth factors and conditions that were not specifically tested here.

In our previous study [26] the specific activity value of the N-terminal Fd-HydA1 fusion was 2800, whereas HydA1 alone was ∼7-fold lower, or 400 U. By applying the purification protocol developed here, we showed an increase from 2800 to 3500 U in the MV activity assay for Fd-HydA1 (25% increase), which declined 3.5-fold to 1000 U after proteolytic removal of the Fd. We speculate that the effect of Fd (the decline in activity by cleavage, or in the opposite case the increase due to the presence of Fd in the fusion) is due to a kinetic advantage from the lower *K*_M_ of Fd (∼300-fold lower than for reduced MV [30]) for HydA1, which effectively increases the rate of MV reduction of HydA1 in Fd-HydA1 versus HydA1 alone. Nonetheless, the HydA1 activity value of 1000 U was still 60% higher than our previously reported value of 400 U. Thus, both the presence of Fd, and revised purification protocol, resulted in isolation of HydA1 with higher specific activity. We hypothesize that fusion of Fd to the HydA1 N-terminus enhanced either the maturation (possibly through reductive activation following H-cluster insertion) or the stability (by maintaining HydA1 in a reduced state in the presence of NaDT) to result in HydA1 with improved specific activities. Finally, we showed that the presence of Fd also improved enzyme recovery during purification by lowering the overall pI to result in higher affinity on DEAE during the ion-exchange step.

The quality of the purified HydA1 was determined in two ways. First, the specific activity was 1000 U based on the MV dependent hydrogen evolution assay. Second the iron incorporation per HydA1 as mol iron (mol HydA1)^−1^ was found to be 5.64+/−0.28, or 94%. Unfortunately, the specific activity level of 1000 U was not maintained during prolonged storage at −80°C in an anaerobic gas-pak jar, after several months the activity values typically declined to a range of 300–400 U. The same HydA1 samples were studied by FTIR spectroscopy and also showed evidence of an exogenous CO absorption peak indicative of the CO-inhibited H-cluster (data not shown) [32]. Thus, under these storage conditions, the loss in activity correlates with the formation of the CO-inhibited form of HydA1. Formation of the CO inhibited HydA1 in frozen samples has been observed by other groups (see [32], [33]), and would arise from H-cluster decomposition and release of CO, which in turn binds to a second enzyme. The net result would be a loss in specific activity of the enzyme pool (from 1000 U to 400 U) due to enzyme degradation.

In conclusion, we have developed an improved recombinant expression and the purification process in *E. coli* for algal [FeFe]-hydrogenase, and for the [FeFe]-hydrogenases in general. Moreover, the basis for the optimization in both the expression and purification steps is not specific for [FeFe]-hydrogenases, and might be useful for improving the recombinant expression and purification of other metallo-enzymes.

## Methods

### Plasmid construction

Plasmid constructs for the expression of *C. reinhardtii* [FeFe]-hydrogenases (HydA1) in *E. coli* were developed from the pETDuet based plasmids pHydEHydA1 and pHydFHydG as described [24]. Modifications to pHydEHydA1 exchanged the *C. reinhardtii* HydA1 structural gene without any other alterations of HydE. To create the N-terminal Fd-HydA1 fusion, the nucleotide sequence encoding the mature form of [FeFe]-hydrogenase HydA1 (Ala57-Lys497) was fused to C-terminus of the mature form of the *C. reinhardtii* PetF (Ala32-Tyr126) (referred to here as Fd) with a 15 amino acid (aa) linker containing a TEV protease recognition site (Thr-Gly-Gly-Gly-Ser-Glu-Asn-Leu-Tyr-Phe-Gln-Gly-Gly-Ala-Ser). This gene constructs was optimized for *E. coli* expression and synthesized by GeneArt. The synthetic Fd-HydA1 gene was sub-cloned into NdeI, AvrII digested pHydEHydA1 replacing the existing *HYDA1* gene with the Fd-HydA1 fusion This construct also contained a C-terminal extension (5′-ctcgagattgaaggccgtcaattgggctggagccatccgcagtttgaaaaataacctagg-3′) encoding the Factor Xa and StrepII-tag (Figure 1).

### MBP-TEV protease expression and purification

To express and purify TEV protease, an expression plasmid containing a gene for the TEV protease fused to Maltose binding protein (MBP) was used. The original TEV-MBP fusion was developed by Kapust *et al.* [28], and later cloned under control of the arabinose promoter by the Benhar group at Tel Aviv University (Prof. I. Benhar *personal communication*). Expressions were performed in *E. coli* BL21 (DE3) Rosetta-2 (Novagen). A 10 ml volume of Luria-Bertani (LB) media containing 200 µg ml^−1^ Carbenicillin was cultured overnight at 37°C. 100 µl of culture was diluted into 10 ml of LB with the same concentration of antibiotics and cultured at 37°C with shaking at 250 rpm until the OD_600_ reached 0.4. 5 ml of the pre-culture was added to 0.5 L of fresh LB media and cultured until the OD_600_ reached a value of 0.8. Cells were induced by addition of 0.2% arabinose and cultured for an additional 4 h at 30°C and 150 rpm.

Cells were collected by centrifugation at 7000×g for 7 min at 4°C, and re-suspended in a 15 ml volume of buffer A (Table 2) without dithionite or glycerol. Cells were disrupted in a French Press for 1 min at 1200 psi. The cell debris was removed by centrifugation at 45,000 rpm in a 60Ti rotor (Beckman) for 30 min at 4°C. Clarified lysates (usually 30–40 ml) were loaded on 15 ml amylose resin columns (NEB) pre-equilibrated with buffer A. The column was washed with 50 ml of buffer A, and elution was carried out in buffer A containing 10 mM maltose. About 30 mg of MBP-TEV was obtained from 0.5 L of culture.

**Table 2:** Buffers used for protein purification.

Buffer	Application step	Composition
A	Cells lysis	Tris-HCl 100 mM, pH 8; glycerol 5% v/v; NaDT 20 mM; protease inhibitor (Sigma) 250 µl L^−1^ of culture; lysozyme 84 µg ml^−1^; benzonase (Sigma) 5 µl L^−1^ of culture
B	1^st^ wash buffer for DEAE	Tris-HCl 100 mM, pH 8; glycerol 5% v/v; NaDT 20 mM
C	2^nd^ wash buffer for DEAE	Tris-HCl 100 mM, pH 8; glycerol 5% v/v; NaDT 20 mM; NaCl 0.05 M
D	DEAE elution buffer	Tris-HCl 100 mM, pH 8; glycerol 5% v/v; NaDT 20 mM; NaCl 0.5 M
E	StrepTactin wash buffer	Tris-HCl 100 mM, pH 8; glycerol 5% v/v; NaDT 20 mM; NaCl 1 M
F	StrepTactin elution buffer	Tris-HCl 100 mM, pH 8; glycerol 5% v/v; NaDT 20 mM; desthiobiotin 12.5 mM
G	Methyl viologen activity assays	Tris-HCl 100 mM, pH 8; glycerol 5% v/v; KCl 0.25 M; triton X-100 0.2% v/v for whole-cell assays.

### Fd-HydA1 expression

All expressions were performed in *E. coli* BL21 (DE3) Rosetta-2 (Novagen). A 10 ml volume of LB containing 200 µg ml^−1^ Carbenicillin and 50 µg ml^−1^ Streptomycin was cultured overnight at 37°C. 100 µl of cells were diluted into 10 ml of Terrific-Broth (TB) with the same concentration of antibiotics and cultured at 37°C with shaking at 250 rpm until absorbance value at OD_600_ reached 0.4. 10 ml of the pre-culture was added into 1 L of fresh TB media in baffled plastic flasks (VWR) and cultured to an OD_600_ value of 0.4. For induction, the culture was transferred to 1 L bottles (Kimax-25) fitted with a V-shape rubber cap and the following supplements were added: fresh ammonium ferric citrate (2.5 mM final), IPTG (1.5 mM final), sodium fumarate (25 mM final), glucose (0.5% final), and cysteine (2 mM final). The bottles were sparged with argon for 1 h at 22°C, after which cultures were transferred to an anaerobic glove box (Coy Laboratories) under 4% H_2_ (balance N_2_) atmosphere and incubated over night at 20°C.

### Fd-HydA1 purification

Due to the oxygen sensitivity of HydA1, all buffers contained 20 mM NaDT. Cells were collected by centrifugation at 7000×g for 7 min at 4°C, and were re-suspended in a 15 ml volume of buffer A (Table 2). Cells were disrupted by Ultrasonication at 5×30 s intervals, and the lysate centrifuged at 45,000 rpm in a 60Ti rotor (Beckman) for 30 min at 4°C. Clarified lysates (usually 30–40 ml) were loaded onto a series of 3×5 ml DEAE FF columns (GE Healthcare) pre-equilibrated in buffer B at a flow rate of 5 ml min^−1^. The column was washed at 5 ml min^−1^ first with 50 ml of buffer B, followed by 50 ml of buffer C. Elution was carried out in buffer D at a flow rate of 2.5 ml min^−1^. The pooled brown fractions (usually 7–10 ml) were combined and digested by addition of 4 ml of MBP-TEV protease (1 mg ml^−1^) for 1 h at 37°C. The NaCl concentration was adjusted to 1 M, and the digested enzyme pool was loaded at 1 ml min^−1^ onto a series of 4×5 ml StrepTactin columns (GE Healthcare) pre-equilibrated and washed in 50 ml of buffer E. HydA1 was eluted in buffer F and 1 ml fractions collected. Protein concentrations were quantified by Bradford assay (Bio-Rad) according to manufacturer instructions. Purified enzymes were stored at −80°C in an anaerobic gas-pak jar (Mitsubishi).

### Protein gel electrophoresis

Protein gel electrophoresis was performed with NuPAGE® Novex® Bis-Tris Gel Systems (Invitrogen) according to manufacturer instructions.

### Western blot

Samples were prepared by dilution of 30 µl of cell lysate into 10 µl LDS sample buffer and 4 µl reducing agent (Invitrogen), and incubated at 95°C for 5 min. A 10 µl volume of the reduced sample were resolved on Novex 10% Bis-Tris SDS-PAGE gels (Invitrogen), and transferred to a 0.45 µm nitrocellulose membrane in an iBlot (Invitrogen). Blots were blocked overnight in PBS containing 3% BSA and 0.5% Tween-20, and washed three times with PBST. Washed blots were incubated 1 h with Mouse Anti Strep-tag® Antibody-Horse Radish Peroxidase conjugate (Pierce) diluted in 1∶10 in PBS containing 0.2% BSA, 0.5% Tween-20, and further diluted 1∶2500 in PBST. Strep-tagged proteins were visualized using the ECL-Plus Kit (GE Healthcare) and western blot images were captured using a Fluor Chem gel documentation system (Alpha Innotech).

### Hydrogen production assays of solubilized *E. coli* whole-cells and purified hydrogenase

Reaction buffer stock solutions were prepared in the anaerobic glove box in a 30 ml sealed serum vial. Methyl viologen (Sigma) was added with NaDT (20 mM final) to 25 ml of buffer G (Table 2) to working concentrations of 20 mM, or 40 µM respectively. The MV reaction buffer was sparged under argon for 20 min. Each sample (10 µl) was diluted into 1 ml of buffer G and added to a stoppered 13 ml serum vial. Sample solutions were sparged with argon for an additional 5 min, and 1 ml of the reaction buffer (MV or Fd) was injected to the sample using a gas-tight syringe. Reactions were mixed and then incubated at 37°C for 10–20 min. The headspace hydrogen was measured by gas chromatograph (GC, Hewlett-Packard 5890 Series II) fitted with a 5 Å mol sieve column (Supelco). 1 unit (U) of hydrogen evolution activity is equivalent to 1 umol hydrogen evolved min^−1^ mg^−1^ for purified samples, and 1 umol hydrogen evolved min^−1^ (ml of culture)^−1^ for cell-free extracts.

### Iron content measurement

Spectrophotometric determination of iron content in purified HydA1 samples was performed as described by Fish [31].
